# Linking diffuse radiation and ecosystem productivity of a desert steppe ecosystem

**DOI:** 10.7717/peerj.9043

**Published:** 2020-05-05

**Authors:** Cheng Li, Xin Jia, Jingyong Ma, Peng Liu, Ruizhi Yang, Yujie Bai, Muhammad Hayat, Jinglan Liu, Tianshan Zha

**Affiliations:** 1 Yanchi Research Station, School of Soil and Water Conservation, Beijing Forestry University, Beijing, China; 2 Key Laboratory of State Forestry Administration on Soil and Water Conservation, Beijing Forestry University, Beijing, China; 3 School of Ecology and Nature Conservation, Beijing Forestry University, Beijing, China

**Keywords:** Diffuse radiation, Clearness index, Gross ecosystem production, Desert steppe ecosystem

## Abstract

Radiation components have distinct effects on photosynthesis. In the desert steppe ecosystem, the influence of diffuse radiation on carbon fixation has not been thoroughly explored. We examined this diffusion and its effect on ecosystem productivity was examined during the growing season from 2014 to 2015 on the basis of eddy covariance measurements of CO_2_ exchange in a desert steppe ecosystem in northwest China. Our results indicated that the gross ecosystem production (GEP) and diffuse photosynthetically active radiation (PAR_dif_) peaked when the clearness index (CI) was around 0.5. The maximum canopy photosynthesis (P_max_) under cloudy skies (CI < 0.7) was 23.7% greater than under clear skies (CI ≥ 0.7). When the skies became cloudy in the desert steppe ecosystem, PAR_dif_ had a greater effect on GEP. Additionally, lower vapor pressure deficits (VPD ≤ 1 kPa), lower air temperatures (T_a_ ≤ 20 °C), and non-stressed water conditions (REW ≥ 0.4) were more conducive for enhanced ecosystem photosynthesis under cloudy skies than under clear skies. This may be due to the comprehensive effects of VPD and T_a_ on stomatal conductance. We concluded that cloudiness can influence diffuse radiation components and that diffuse radiation can increase the ecosystem production of desert steppe ecosystems in northwest China.

## Introduction

Gross ecosystem production (GEP), the overall photosynthetic fixation of carbon per unit of space and time (*Monteith, 1972; Monteith, 1977*), is the determining factor in biogeochemical cycles of terrestrial ecosystems (*Lin et al., 2017*). The primary influence on daytime carbon uptake in terrestrial ecosystems during the growing season is solar radiation (Guan et al., 2006; Bai et al., 2012). The incoming solar radiation’s quantity and quality composition can affect GEP (*Gu et al., 2003; Bai et al., 2012*). Previous studies have indicated that atmospheric cloud content changes can affect solar radiation on the ground surface and can balance the direct and diffuse solar radiation components (*Gu et al., 2003; Niyogi et al., 2007; Urban et al., 2007; Zhang et al., 2011a; Zhang et al., 2011b; Gao et al., 2018*). Since plant canopies can more efficiently use diffuse radiation for photosynthesis than direct radiation, increased diffuse radiation under cloudy sky conditions can significantly enhance GEP (*Goudriaan, 1977; Gu et al., 2002; Farquhar, Roderick & Pinatubo, 2003; Gu et al., 2003; Alton, North & Los, 2007b; Mercado et al., 2009; Tong et al., 2017*). Under cloudy sky conditions, the total and direct radiation received by the canopy decreases, while the amount of diffuse radiation increases. The increased diffuse radiation more evenly distributes radiation throughout the canopy (*Roderick et al., 2001; He et al., 2013*), improving leaf photosynthesis (*Urban et al., 2007; Knohl & Baldocchi, 2008; Mercado et al., 2009; Still et al., 2009*). Therefore, canopy photosynthesis under cloudy skies with more diffuse radiation was more effective than under clear skies (*Gu et al., 2002; Farquhar, Roderick & Pinatubo, 2003; Alton, North & Los, 2007b; Mercado et al., 2009; Tong et al., 2017*). How photosynthesis responds to different sky conditions in forests (*Gu et al., 1999; Rocha et al., 2004; Dengel & Grace, 2010; Zhang et al., 2010; Urban et al., 2012; Xu et al., 2018; Park et al., 2018*) and grassland ecosystems (*Jing et al., 2010; Zhang et al., 2011b; Bai et al., 2012*) has been researched in previous studies. Due to smaller leaf area index values and the weaker photosynthesis abilities for low canopy ecosystems, the positive effects of diffuse radiation may be reduced (*Letts, Lafleur & Roulet, 2005*) or eliminated (*Kanniah, Beringer & Hutley, 2013*). However, other studies have found that diffuse radiation enhances the carbon sink in grassland ecosystems (*Zhang et al., 2011b; Bai et al., 2012*). A previous study of 23 sampled sites (including grasslands, farmlands, and forests) found that light use efficiency can be improved under high diffuse radiation condition, but it was not significantly different across vegetation types in the great plains of the southern United States (*Wang, Dickinson & Liang, 2008*). Ultimately, our understanding of diffuse radiation on ecosystem carbon fixation remains limited because the effects differ across vegetation types. Additionally, diffuse radiation interacts with other environmental factors, such as temperature and moisture conditions (*Cheng et al., 2015*).

With increases in climate change and variability, China has seen a rising trend in diffuse radiation (*Mercado et al., 2009; Ren et al., 2013*). Previous studies have shown that China’s diffuse radiation increased by 7.03 MJ m^−2^ yr^−1^ annually from 1981 to 2010 (*Ren et al., 2013*). Over the past 15 years, Northwest China’s cloud coverage has reduced, but the cloud optical depth has increased (*Ren et al., 2013*). The question now is how diffuse radiation affects the GEP of the desert steppe ecosystem, and whether cloudiness positively or negatively affects the GEP.

We hypothesized that diffuse radiation positively affects a steppe ecosystem’s GEP under different environmental conditions. We used the eddy covariance technique to measure the CO_2_ exchange and to examine the effect of diffuse radiation on the GEP in a desert steppe ecosystem at the southern edge of the Mu Us desert from 2014 to 2015. Our research aimed to determine the relationship between diffuse radiation and canopy productivity and to examine whether cloud-induced changes in radiation components enhance canopy productivity. Our objectives were to: (1) quantify the effect of cloudiness on the light response process, (2) examine how GEP changes with diffuse radiation, and (3) explore the relationship between environmental factors and GEP under different sky conditions.

## Material and Methods

### Site description

**Figure 1:**
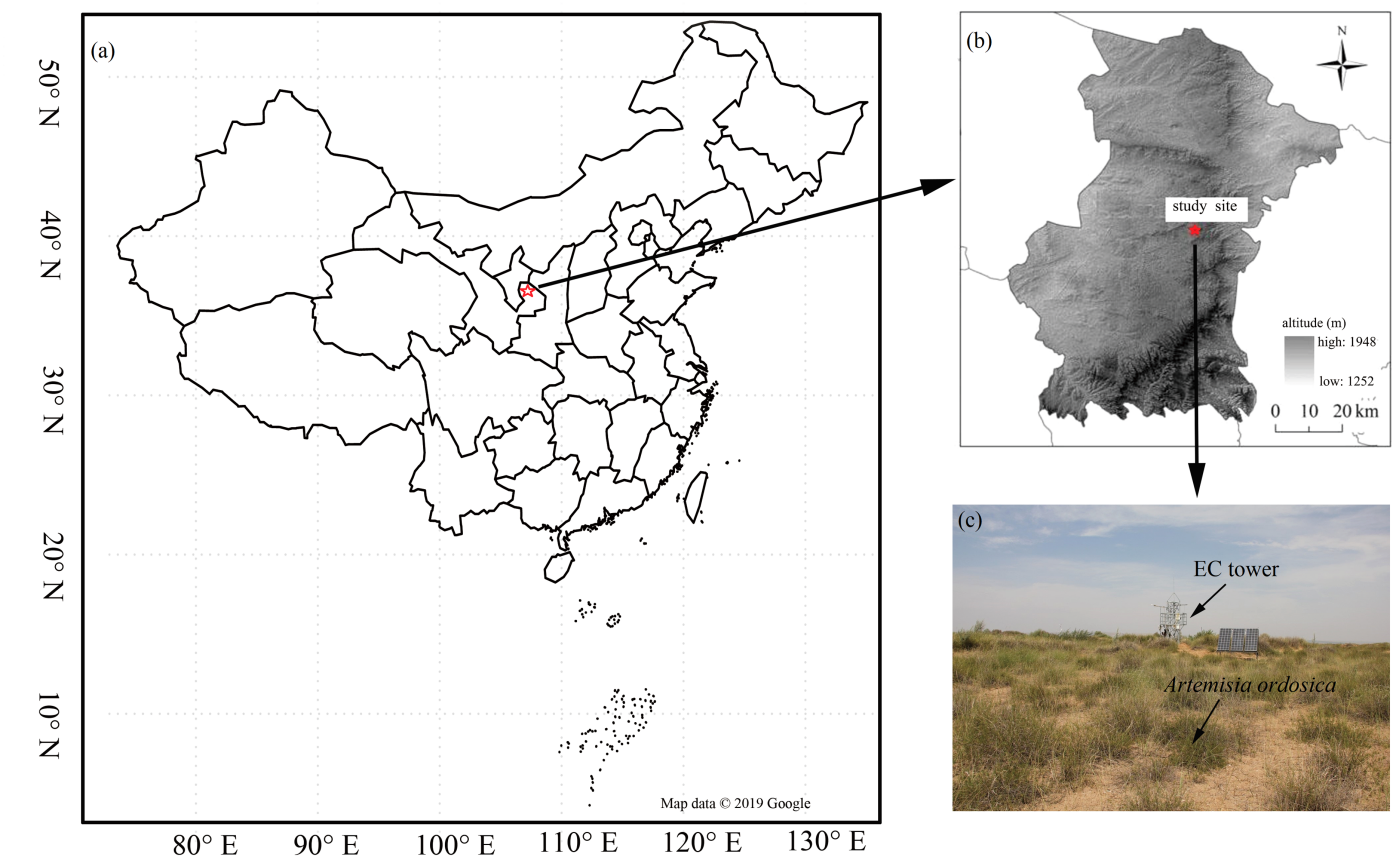
The distribution of the study flux site. The location of the study flux site (A) and (B), and vegetation condition (C). Map data ©2019 Google.

Our research was conducted in a desert steppe ecosystem on the southern edge of the Mu Us desert (37°42′31″N, 107°13′47E, 1,560 m above sea level) in Ningxia, Northwest China (Fig. 1). The long-term mean air temperature (1954–2004) was 8.1 °C and the area is frost-free for an average of 165  days a year (*Wang et al., 2014*). Annual precipitation is 287 mm, 62% of which falls between July and September (*Feng et al., 2013; Jia et al., 2014*). Annual incoming shortwave radiation is 1. 4 ×10^5^ J cm^−2^ (*Gong et al., 2016*), and mean annual potential evapotranspiration is 2,024 mm (*Jia et al., 2014*). The site’s ground is flat with a homogeneous underlying surface. Vegetation is dominated by C_3_ herbaceous plants: *Leymus secalinus*, *Pennisetum flaccidum*, *Stipa breviflora*, *Cleistogenes squarrosa*, and a scattered encroachment of the C_3_ shrub species *Artemisia ordosica*.

### CO_2_ flux and meteorological measurements

We used an eddy covariance (EC) system, which we mounted at a 4.2 m height above the ground, to measure the CO_2_ exchange between vegetation and atmosphere (*Xie et al., 2015*). The EC system consisted of a closed-path infrared gas analyzer (IRGA; model LI-7200, LI-COR Biosciences, Lincoln, NE, USA) and a sonic anemometer (WindMaster™ Pro, Gill Instruments Ltd, Lymington, England). We used the gas analyzer to measure fluctuations in CO_2_ and water vapor concentrations. The anemometer was used to measure fluctuations in wind speed, direction, and sonic temperature. We calibrated the LI-7200 every three months using 99.99% nitrogen gas to calibrate zeros for both CO_2_ and water vapor, and a 650 ppm CO_2_ standard and a dew point generator (LI-610, LI-COR Inc., USA) to calibrate the span for CO_2_ and water vapor, respectively. Wind speed, sonic virtual temperature, CO_2_ concentrations, and H_2_O concentrations were sampled at 10 Hz, and the data were stored in a data logger (LI-7550, LI-COR Inc., USA). Additionally, due to the short length of the canopy (1.4 m), the canopy’s CO_2_ storage was not used to estimate net ecosystem production (NEP) (*Zhang et al., 2007*). The CO_2_ storage term tended to be close to zero when using daily and annual time scales (*Baldocchi, 2003; Jia et al., 2014*).

Simultaneously, we measured above-canopy meteorological variables at the top of the tower each half hour. Incident photosynthetically active radiation (PAR in µmol m^−2^ s^−1^) was measured using LI-190SA quantum sensors (LI-COR Biosciences, Lincoln, NE, USA). Air temperature (T_a_ in^∘^ C) and relative humidity (RH) were measured using a thermohygrometer (HMP155A, Vaisala, Finland). The vapor pressure deficit (VPD) was calculated accordingly from T_a_ and RH. Precipitation (mm) was determined using a tipping bucket rain gauge (TE-525 MM, Campbell Scientific, UT, USA). Soil temperature (T_s_ in^∘^ C) and volumetric soil water content (SWC) profiles at 10 cm depths below ground adjacent to the EC tower were monitored using ECH_2_O-5TE sensors (Decagon Devices Inc., Pullman, WA, USA). We stored all meteorological variables in data loggers (CR200X and CR3000, Campbell Scientific Inc., USA).

### Data processing

We used the post-processing software Eddypro 4.0.0 to compute flux covariance from the raw data. The mean covariance between fluctuations in vertical wind speed and CO_2_ concentration was determined by the net ecosystem CO_2_ exchange (NEE, µmol CO_2_ m^−2^ s^−1^) flux between the ecosystem and atmosphere. Downward fluxes values were considered as negative and upward fluxes values as positive. Our calculations included a 3-D coordinate rotation, spike detection, and checks for instantaneous records exceeding realistic absolute limits. In addition, we used air density fluctuations to correct the CO_2_ fluxes (*Webb, Pearman & Leuning, 1980*). Nighttime data with friction velocities below the 0.18 m s^−1^ threshold were removed to eliminate underestimating stable stratification (*Jia et al., 2014*). The friction velocity threshold was estimated following the China FLUX standard method (*Zhu et al., 2006*).

We chose a gap-filling model based on the magnitude and bias of residuals and the stability of model-parameter estimates (Xie et al., 2016). Small gaps (≤2 h) were filled using linear interpolation, while larger gaps were filled during the night (PAR <5 µmol m^−2^ s^−1^) according to a Q_10_-model (*Xie et al., 2015*):

(1) \begin{eqnarray*}NE{E}_{\mathrm{night}}=R{e}_{10}\times {Q}_{10}^{({T}_{S}-10)/10}\end{eqnarray*}

where *NEE*_night_ is the nighttime NEE, *T*_*s*_ is the soil temperature at a 10-cm depth, *Re*_10_ is ecosystem respiration at *T*_*s*_ = 10 °C, and *Q*_10_ is the temperature sensitivity of *Re*.

Daytime ecosystem respiration ((*Re*) was estimated from the nighttime NEE-based calibration of Eq. (1) using monthly values, assuming consistent temperature sensitivity between nighttime and daytime exchanges. NEP was estimated as—NEE. Once daytime estimates of *Re* were available, gaps in daytime NEE were filled with:

(2) \begin{eqnarray*}\mathrm{NEE}=\mathrm{Re}- \frac{{\alpha }^{{^{\prime}}}\times PAR\times {P}_{{\max \nolimits }^{{^{\prime}}}}}{{\alpha }^{{^{\prime}}}\times PAR+{P}_{max}} .\end{eqnarray*}

where *α*′ is the apparent quantum yield (µmol CO_2_ µmol PAR^−1^), PAR is photosynthetically active radiation in µmol m^−2^ s^−1^, and *P*_max′_ is the maximum apparent photosynthetic capacity of the canopy (µmol CO_2_ m^−2^ s^−1^). Off-season Re was considered as 24 h NEE fluxes. GEP was estimated as:

(3) \begin{eqnarray*}GEP=Re+NEP\end{eqnarray*}

The gaps in vapor pressure deficit (VPD, kPa) were filled from the gap-filled T_a_ and RH records above the canopy. T_s_ and T_a_ value gaps were filled using the mean diurnal variation (MDV) method (*Jia et al., 2016*), while missing PAR values were empirically filled with half-hourly PAR data from a meteorological tower about 3 km east. Other 30-min gaps <1.5 h in meteorological variables with underlying diurnal cycles were filled using linear interpolation, and longer gaps (i.e., gaps ≥ 1.5 h) were filled using the Mean Diurnal Variation method (*Falge et al., 2001*).

Relative extractable soil water (REW) was calculated using Eq. (4) below, with REW <0.4 and REW ≥ 0.4 representing soil water stress and non-stress, respectively (*Granier et al., 1999*).

(4) \begin{eqnarray*}REW= \frac{SWC-SW{C}_{\min }}{SW{C}_{\max \nolimits }-SW{C}_{\min \nolimits }} \end{eqnarray*}

where *SWC*_max_ and *SWC*_min_ are the maximum and minimum soil water content measured at 10 cm depths, respectively.

### Defining diffuse PAR and sky conditions

Diffuse PAR was calculated based on the clearness index (CI) and solar elevation angle (*β*) (*Gu et al., 1999*). The corresponding equations were as follows:

(5a) \begin{eqnarray*}& & PA{R}_{dif}=PA{R}_{tot} \frac{[1+0.3(1-{q}^{2})]q}{1+(1-{q}^{2}){\cos \nolimits }^{2}(9{0}^{\circ }-\beta ){\cos \nolimits }^{3}\beta } \end{eqnarray*}

(5b) \begin{eqnarray*}& & \mathrm{q}=({S}_{f}/{S}_{e})/\mathrm{CI}\end{eqnarray*}

When 0 ≤ CI ≤ 0.3, constraint: *S*_*f*_∕*S*_*e*_ ≤ CI,

(5c) \begin{eqnarray*}{S}_{f}/{S}_{e}=CI(1.020-0.254CI+0.0123 \sin \nolimits \beta )\end{eqnarray*}

When 0.3 <CI ≤ 0.78, constraint: 0.1CI ≤ *S*_*f*_∕*S*_*e*_ ≤ 0.97CI,

(5d) \begin{eqnarray*}{S}_{f}/{S}_{e}=CI(1.400-1.749CI+0.177\sin \nolimits \beta )\end{eqnarray*}

When 0.78 <CI, constraint: *S*_*f*_∕*S*_*e*_ ≥ CI,

(5e) \begin{eqnarray*}{S}_{f}/{S}_{e}=CI(0.486CI-0.182 \sin \nolimits \beta )\end{eqnarray*}

where *PAR*_*tot*_ is the total PAR, *PAR*_*dif*_ is the diffuse PAR, and *S*_*f*_ is the total diffuse radiation received by the horizontal plane of the Earth’s surface (W m^−2^).

CI is defined as the ratio of the global solar radiation (S) received at the Earth’s surface to the extraterrestrial irradiance (Se) in a plane parallel to the Earth’s surface (*Gu et al., 1999*), such that:

(5f) \begin{eqnarray*}& & CI= \frac{S}{{S}_{e}} \end{eqnarray*}

(5g) \begin{eqnarray*}& & {S}_{e}={S}_{sc}[1+0.033cos(360{t}_{d}/365)] \sin \nolimits \beta \end{eqnarray*}

(5h) \begin{eqnarray*}& & \mathrm{Sin}\beta =\sin \nolimits \varphi \ast \sin \nolimits \delta +cos\varphi \ast \cos \nolimits \delta \ast \cos \nolimits \omega \end{eqnarray*}

where *S*_*sc*_ is the solar constant (1,368 W m^−2^), *t*_*d*_ is the day of the year, *β* is the solar elevation angle, *φ* is the degree of latitude, *δ* is the declination of the sun, and *ω* is the time angle.

CI has been commonly used to indicate sky conditions (*Rocha et al., 2004; Letts, Lafleur & Roulet, 2005; Urban et al., 2007; Alton, 2008; Knohl & Baldocchi, 2008*). To analyze how diffuse PAR responds to GEP under different sky conditions, we defined the two sky conditions as clear sky (CI ≥ 0.7) and cloudy sky (CI <0.7). The cloudy sky condition was divided into thin clouds (0.3 < CI <0.7) and thick clouds (CI ≤ 0.3) (*Chen, Shen & Kato, 2009*).

### Diffuse light response parameters

We estimated photosynthetic parameters from light-response curves as described by the Michaelis–Menten equation below:

(6) \begin{eqnarray*}\mathrm{GEP}= \frac{\alpha \times {P}_{\max }\times PA{R}_{dif}}{\alpha \times PA{R}_{dif}+{P}_{\max }} \end{eqnarray*}

where GEP is gross ecosystem production, *PAR*_*dif*_ is diffuse PAR, *α* is ecosystem apparent quantum yield as an initial slope of light response curve of photosynthesis, and *P*_*max*_ is maximum photosynthesis.

### Statistical analyses

We fit Eq. (6) using GEP data collected each half hour from June to August to evaluate the variations in light response. The regressions were conducted on bin-averaged data using a PAR interval of 50 µmol m^−2^ s^−1^. In order to test the dependency of the GEP_day_-PAR relationship on different environmental factors, and to exclude the influences of plant phenology, we compiled GEP_day_ data from the mid-growing season into multiple groups. The daytime data from 10:00 to 16:00 in the mid-growing season (June-August, inclusive) was used for this analysis because plant photosynthesis is most active during this period, and the influence of the solar altitude angle on CI could be eliminated (Bai et al., 2012; Prior, Eamus & Duff, 1997).

In order to further examine how GEP responds to changes in the diffuse PAR, environmental conditions were separated into three T_a_ classes (T_a_ <20 °C, 20 <T_a_ ≤ 25 °C, and T_a_ >25 ° C), three VPD classes (VPD <1 kPa, 1 <VPD ≤ 2 kPa, and VPD >2 kPa) (*Zhang et al., 2011a*), and two water conditions.

We used path analysis in AMOS (version 24.0, Chicago, IL, USA) to examine how environmental factor interactive controls affect GEP under different sky conditions. All selected regression curves were statistically significant (*p* < 0.05) and based on several related studies (*Zhang et al., 2010; Oliphant et al., 2011; Bai et al., 2012; Kanniah, Beringer & Hutley, 2013*) that had been conducted using SPSS software (2012, ver. 22.0; SPSS Inc., USA). The standardized path coefficient (*r*), an analogy of the correlation coefficient, was used to quantify the effect of one variable on another (*Shipley, 2002*). The final model had a high goodness-of-fit when the good fit index (GFI) was greater than 0.9 and the root mean square error of approximation (RMSEA) was less than 0.05 (*Kline, 2011*).

## Results

### Environmental factors and CI

**Figure 2:**
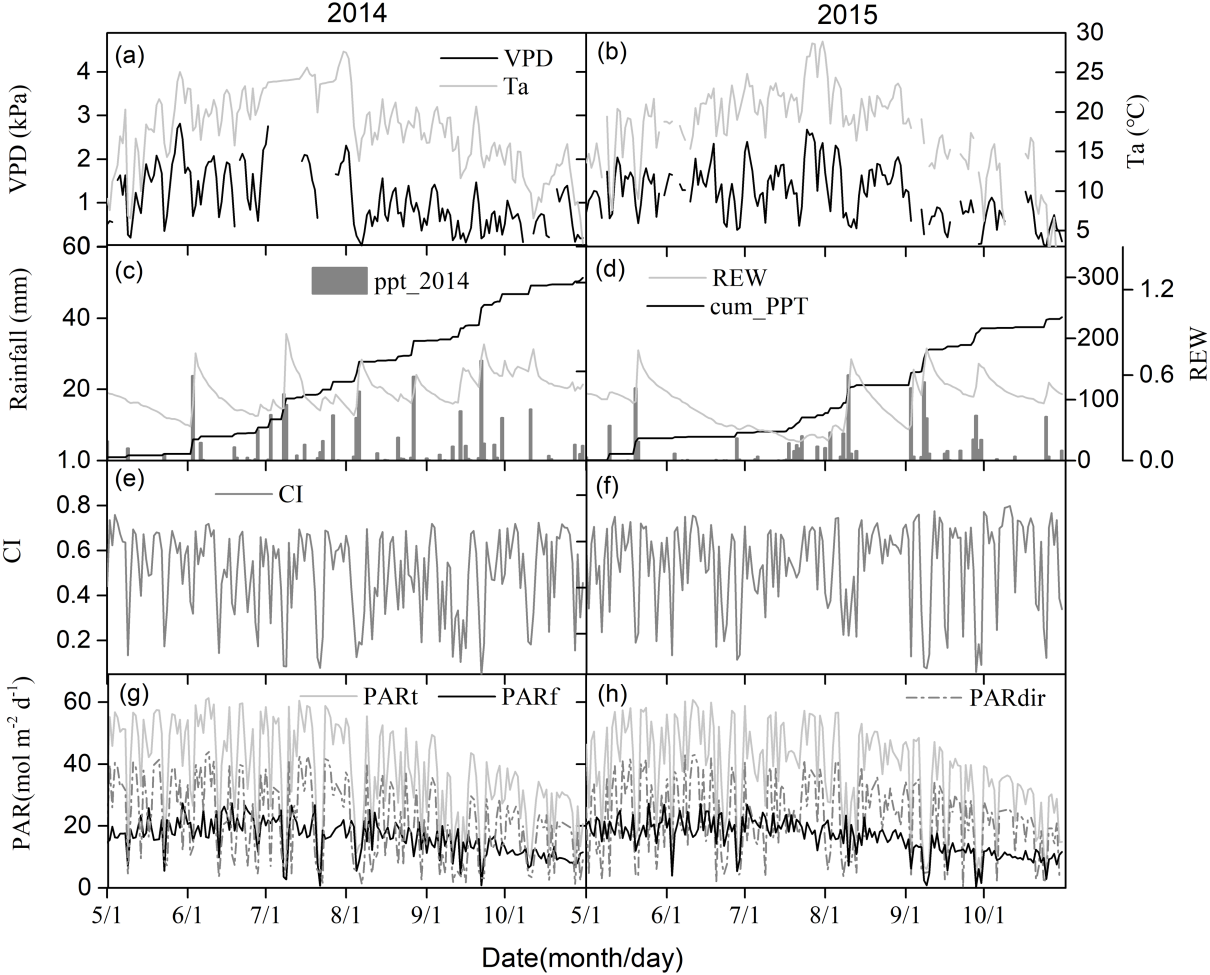
The seasonal variations of environmental factors. (A) and (B) Daily mean air temperature (T_*a*_) at 4.2 m above ground and vapor pressure deficit (VPD), (C) and (D) relative extractable soil water (REW) at 10 cm depth and daily-integrated and cumulative precipitation, (E) and (F) clearness index (CI), (G) and (H) daily-integrated incident total, diffuse and direct photosynthetically active radiation (PAR) in 2014 and 2015 growing season (May–October).

Figures 2A and 2B show seasonal variations in the daily mean air temperature (T_a_) and vapor pressure deficit (VPD). Seasonal patterns in T_a_ and VPD were similar across different years, peaking during the mid-growing seasons. Relative extractable soil water (REW) at 10-cm depths, daily sum precipitation, and cumulative precipitation showed clear seasonal patterns (Figs. 2C and 2D). The total rainfall at the middle of the growing seasons was 185.5 mm and 87.1 mm in 2014 and 2015, respectively, with one big rain event generating more than 20 mm day^−1^ in 2014. Due to uneven rainfall from year to year, plants experienced 23 days of soil water stress (REW < 0.4) in 2014 and 86 days of soil water stress in 2015 (Figs. 2C and 2D).

Seasonal CI variations are shown in Figs. 2E and 2F. 2014 had a similar seasonal pattern as 2015. Changes in incident PAR daily totals were consistent with those of diffuse and direct PAR, with an annual total of 393.2 MJ m^−2^ for incident PAR in 2014, 398.1 MJ m^−2^ for incident PAR in 2015, 168.8 MJ m^−2^ for diffuse PAR in 2014, and 167.0 MJ m^−2^ for diffuse PAR in 2015 (Figs. 2G and 2H).

**Figure 3:**
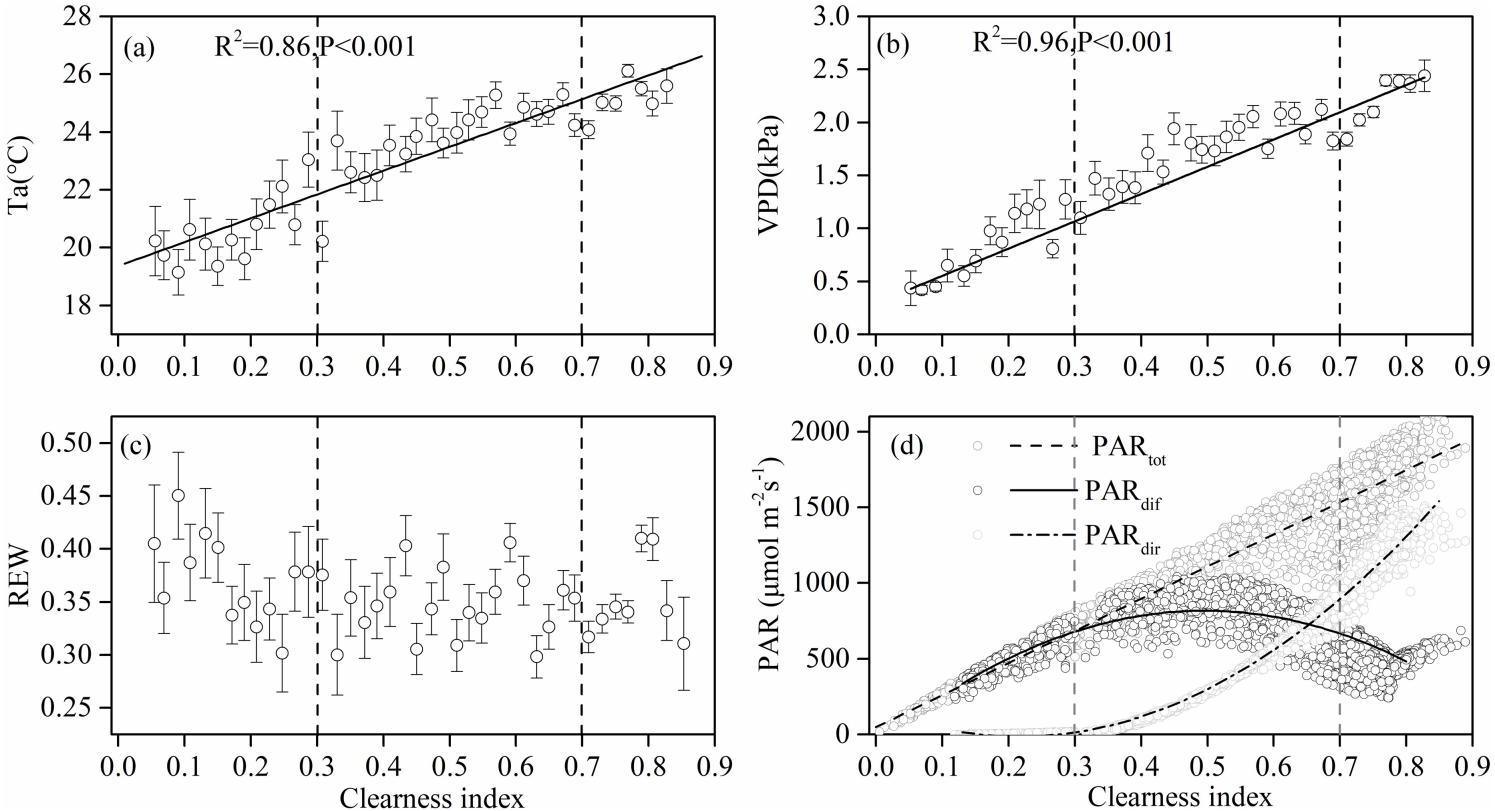
The relationships between clearness index and the environmental factors. Relationships between clearness index and (A) air temperature (T_*a*_) at 4.2 m above ground, (B) vapor pressure deficit (VPD), (C) relative extractable soil water (REW) at 10 cm depths, and (D) total incident photosynthetically active radiation (PAR), diffuse PAR and direct PAR. Data used are half-hourly values between 10 am and 4 pm in mid-growing season (June–August) in 2014 and 2015. Half-hourly data was bin-averaged by CI increment of 0.1. Bars indicate standard errors.

Figure 3 shows the effect of cloudiness on T_a_, VPD, REW, and PAR (including total, direct, and diffuse) measurements during the studied period. T_a_ and VPD increased linearly with an increase in CI (Figs. 3A, 3B), while no obvious relationship was observed between REW and CI (Fig. 3C). Total PAR increased linearly with an increase in CI (Fig. 3D). As the sky became clearer (CI >0.3), direct radiation increased exponentially with CI (Fig. 3D). The diffuse PAR increased as CI increased, peaking at a CI of 0.5 under thin cloud conditions, then decreased as CI continued to increase (Fig. 3D). When CI exceeded 0.8, the diffuse radiation increased.

### Light response parameters and GEP under different sky conditions

**Figure 4:**
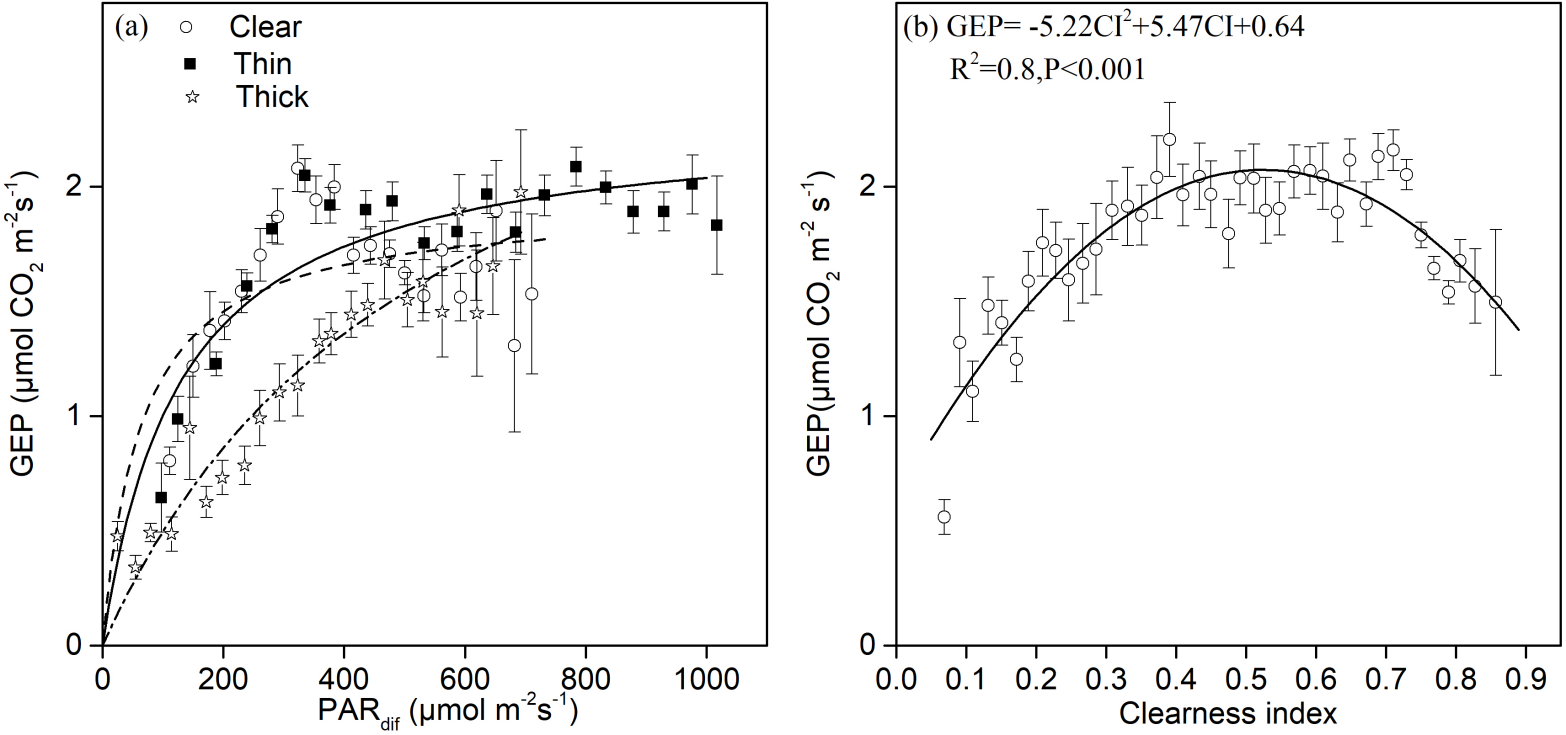
The relationships between different sky conditions and the gross ecosystem production. Daytime gross ecosystem production (GEP) as a function of (A) diffuse photosynthetically active radiation (PAR_*dif*_) and of (B) the clearness index (CI) during mid-growing season (June–August) in 2014 and 2015. Half-hourly GEP was bin-averaged (A) by PAR increment of 50 µmol CO_2_ m^−2^ s^−1^ and (B) by CI increment of 0.1. Bars indicate standard errors.

Light response curves under different sky and environmental conditions are shown in Fig. 4A. We found significant quadratic relationships between the GEP and diffuse radiation under sunny and thin cloud conditions, and an approximately linear relationship under thick cloud conditions. There was a higher potential P_max_ under cloudy conditions with a larger proportion of diffuse radiation than under clear sky conditions. Under clear sky conditions, we observed photo-inhibition phenomena (Fig. 4A). The GEP peaked when CI fluctuated around 0.5 (Fig. 4B).

The light-response curve *α* and P_max_ parameters are listed in Table 1. As the sky became cloudy, the P_max_ became larger and the *α* value became smaller. The P_max_ was 39% and 68% greater under thick clouds than under thin clouds and clear skies, respectively. The *α* value was 400% and 68% greater under clear skies than under thick clouds and thin clouds, respectively. The P_max_ was 23.7% greater under cloudy skies (CI < 0.7) than under clear skies (CI ≥ 0.7), and the *α* value was 122% greater under clear skies than under cloudy skies (Table 1). As the T_a_ and VPD rose, the P_max_ gradually became smaller (Table 1).

**Table 1:** The light-response curve parameters under different environment conditions and different sky conditions. Parameter values describing the response of daytime gross ecosystem production (GEP) to incident diffuse photosynthetically active radiation (PAR_dif_) and incident total photosynthetically active radiation (PAR_tot_) (the last column) between 10 am and 4 pm during mid-growing season (June–August) under different environmental conditions in 2014 and 2015.

Treatment	*α*	P_max_	Adj.R^2^
	µmol CO_2_ µmol photon^−1^	µmol m^−2^ s^−1^	
VPD ≤ 1	0.0162	2.86	0.59
1<VPD ≤ 2	0.0311	2.34	0.39
VPD>2	0.0142	1.99	0.75
T_a_≤ 20	0.0141	3.11	0.88
20<T_a_≤ 25	0.0209	2.46	0.54
T_a_> 25	0.0148	2.27	0.56
REW< 0.4	0.0184	2.22	0.64
REW ≥ 0.4	0.0214	2.37	0.60
Cloudy sky Thin clouds Thick clouds	0.0134 0.0178 0.0059	2.38 2.30 3.23	0.92 0.79 0.89
Clear sky	0.0299	1.93	0.30
Diffuse radiation	0.0102	2.608	0.85
Total radiation	0.0084	2.197	0.74

**Figure 5:**
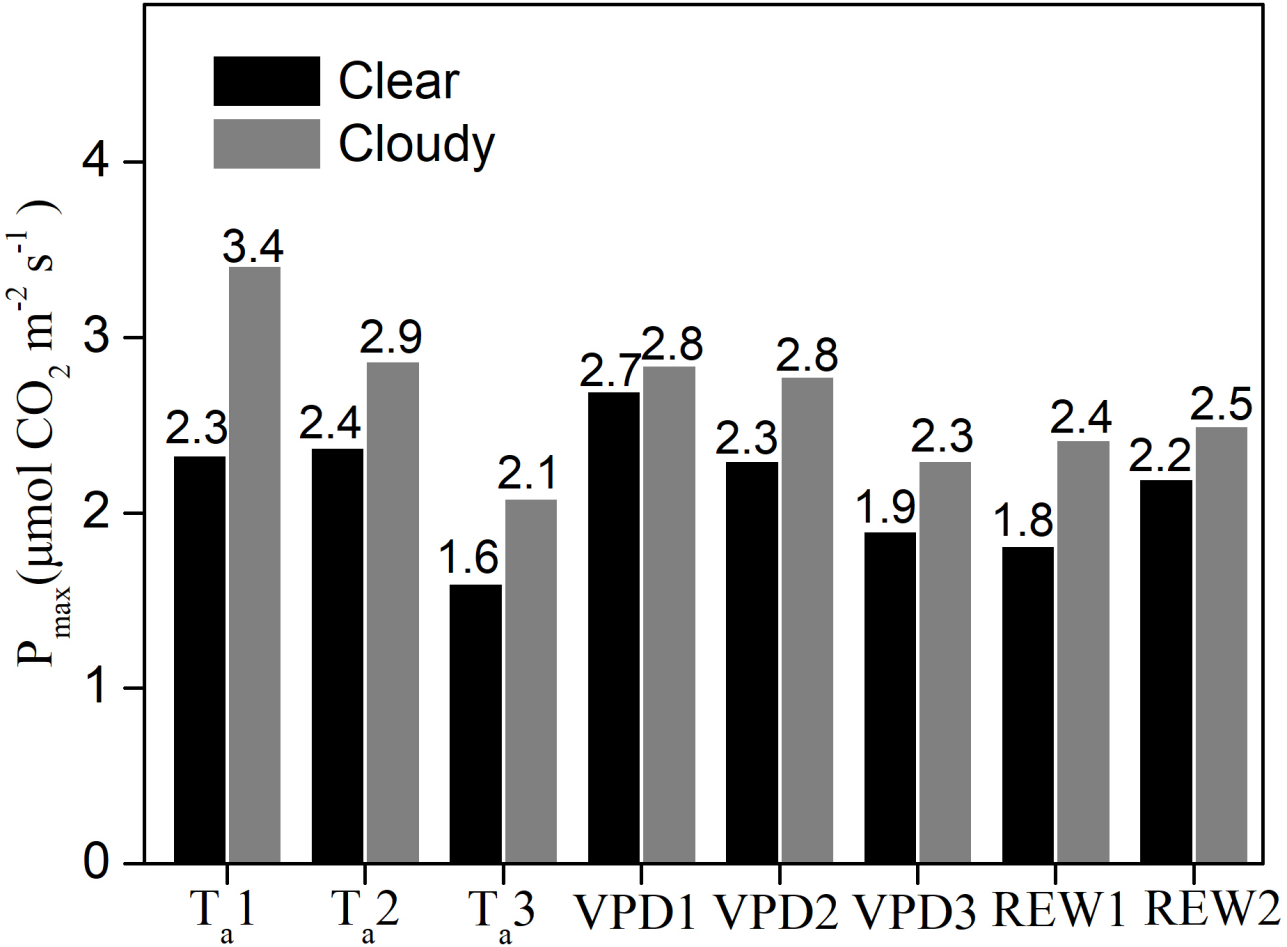
The maximum ecosystem photosynthesis P_max_ under different environment conditions. Maximum ecosystem photosynthesis (P_max_ in µmol CO_2_ m^−2^ s^−1^) in clear skies and cloudy skies under various environmental conditions. Data were fitted using analysis of covariance (ANCOVA). Values above the bars were P_max_ under different condition. T_*a*1_, T_*a*2_ and T_*a*3_ represent air temperature T_a_ ≤ 20 °C, 20 < *T*_*a*_ ≤ 25 °C and T_a_ > 25°C, respectively. VPD1, VPD2 and VPD3 represent vapor pressure deficit VPD ≤ 1 kPa, 1 < VPD ≤ 2 kPa and VPD > 2kPa, respectively. REW1 and REW2 represent relative extractable soil water content REW < 0.4 and REW ≥ 0.4, respectively.

Under similar T_a_, VPD, and water content classes, the P_max_ values were significantly greater under cloudy conditions than under clear skies (Fig. 5). Compared to clear sky conditions, the cloudy condition P_max_ values were 46.5%, 20.7%, and 30.4% higher under the three T_a_ classes; 5.3%, 20.9%, and 21% higher under the three VPD classes; 33% and 13% higher under the two water stressed conditions, respectively. Under cloudy sky conditions, the P_max_ value under T_a_ ≤ 20 °C was 19% higher than that under 20 <T_a_ ≤ 25 °C and 64% higher than that under T_a_ > 25 °C, respectively. Under cloudy sky conditions, the P_max_ value was 2.3% higher under VPD ≤ 1 kPa than under 1 <VPD ≤ 2  kPa, and 24% higher than under VPD > 2 kPa. In cloudy sky conditions, the P_max_ was 3.2% higher under non water-stressed conditions than under water-stressed conditions. The results indicated that lower T_a_, lower VPD, and non-stressed REW accelerated the ecosystem photosynthetic potential under cloudy skies.

### Direct and indirect influences of environmental factors on GEP

**Figure 6:**
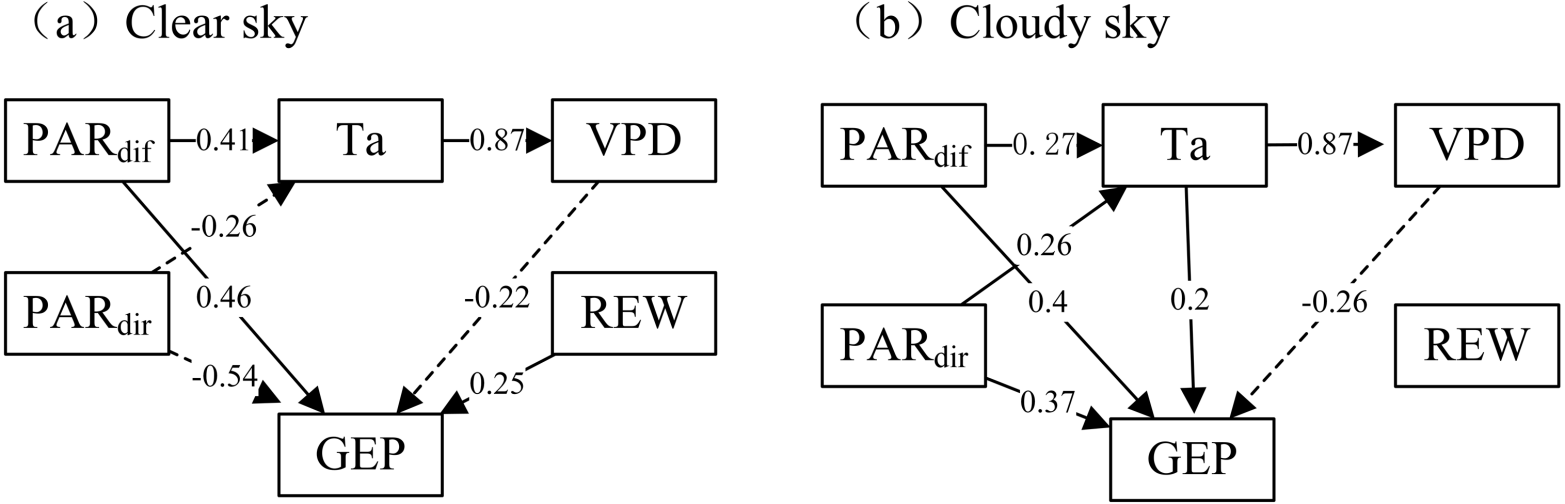
The path analysis diagram. Direct and indirect effects of environmental factors on gross ecosystem production (GEP) under clear sky and cloudy sky conditions during mid-growing season (June–August) in 2014 and 2015. Values in the path figure are standardized path coefficients (PV: –1 to 1). Dashed solid line with PV < 0 denotes negative correlation and solid line with PV > 0 denotes positive correlation.

Based on the path coefficient analysis, our findings further confirmed the direct and indirect influences of PAR_dif_ on GEP. When the sky became cloudy, the PAR_dif_ more positively influenced GEP (Fig. 6). Additionally, the PAR_dir_ positively affected GEP under cloudy conditions and negatively affected GEP under clear sky conditions. In most cases, VPD and T_a_ negatively affected GEP. The influence of VPD on GEP was more negative under cloudy conditions than under clear skies. T_a_ was a weak influence on GEP under cloudy sky conditions. The indirect effect of T_a_ on GEP was negative, indicating that a T_a_ increase would indirectly lead to a GEP decrease via an increasing VPD. REW demonstrated positive effects on GEP under clear skies but no obvious effects under cloudy skies.

## Discussion

### Factors influencing light response parameters

We concluded that the desert steppe had a higher P_max_ under cloudy conditions. Our findings were similar to those in previous studies on crop (Tong et al., 2014) and grassland ecosystems (Jing et al., 2010; Zhang et al., 2011b; Bai et al., 2012; Huang et al., 2014). However, the P_max_ for a tropical savanna ecosystem was found to be highest under clear sky conditions (Kanniah, Beringer & Hutley, 2013). This may be due to a relative lower radiation intensity under cloudy conditions compared with sunny conditions in the tropical savanna. The *α* value in this study is consistent with those in forest ecosystem studies, where the cloudy sky appeared larger than the clear sky (Hollinger et al., 1994; Rocha et al., 2004; Dengel & Grace, 2010; Zhang et al., 2010). This result indicates that the desert steppe ecosystem has a higher potential light use efficiency.

We found higher P_max_ values under cloudy rather than sunny skies for two reasons. First, radiation composition and quality and light distribution variations within an ecosystem influenced the canopy’s photosynthetic productivity under cloudy conditions (*Knohl & Baldocchi, 2008*). Second, cloudy skies lead to a reduction in T_a_ and VPD, which increase canopy stomatal conductance and enhance photosynthetic rates (*Huxman et al., 2003; Xu et al., 2018*).

However, the results of this research are not consistent with other previous studies (*Letts, Lafleur & Roulet, 2005; Niyogi et al., 2007*) that did not find an increase in the photosynthesis of ecosystems with low leaf area indexes, such as grasslands and shrublands, under cloudy conditions. The explanation for this may be that a decrease in GEP under cloudy conditions caused by total radiation reduction is greater than an increase in GEP caused by diffuse fertilization. We attribute this inconsistency to environmental differences in the studied ecosystems, such as local thermal, water, and light conditions (*Zhang et al., 2011b*).

### Interplay of factors on GEP under different sky conditions

Different sky conditions caused radiation composition and other environmental factor variations, leading to corresponding effects on GEP. Our path analysis results showed that in the desert steppe ecosystem under cloudy skies the PAR_dif_ more positively influenced GEP, while T_a_ and VPD suppressed GEP in most cases (Fig. 6). The existence of clouds may be both a cause and consequence of solar radiation, temperature, moisture, precipitation, and other atmospheric factors (*Gu et al., 1999; Zhang et al., 2011a; Zhang et al., 2011b*). These factors have direct and indirect effects on the biophysical processes of ecosystem productivity.

Our study showed that the P_max_ increased under lower T_a_ and VPD conditions. However, under the same environmental factor limitations, VPD reduced P_max_ more significantly than T_a_ under cloudy conditions (Fig. 5). The increase in T_a_ and VPD limited the GEP regardless of sky conditions. Under cloudy sky conditions, T_a_ and VPD had a secondary direct effect on GEP. A decrease in T_a_ can cause decreased ecosystem respiration (*Gu et al., 1999; Urban et al., 2007*). An increase in T_a_ might result in increased VPD, leading to the indirect inhibition of GEP. Increases in VPD can limit GEP under varying environmental conditions, and leaf guard cell’s behavior is highly controlled by the inside and outside vapor pressure balance (*Farquhar & Sharkey, 1982*). On the other hand, higher VPD levels affect stomatal closure, thus controlling photosynthetic rates (*Zhou et al., 2013; Goodrich et al., 2015*).

Under clear sky conditions, sunlit canopy leaves showed an obvious photo-inhibition phenomenon (Fig. 4A), similar to that of an open shrub-land ecosystem (*Keenan et al., 2019*). Higher T_a_ and VPD under clear sky conditions (Figs. 3A, 3B) may cause the stomatal closure of leaf guard cells that are protecting themselves against high radiation (*Zhou et al., 2013; Goodrich et al., 2015*). Previous studies have shown that strong UV radiation, especially ultraviolet-B (UV-B), can inhibit photosynthesis (Ekelund, 2000; Correia et al., 2005; Sangtarash et al., 2009; Li et al., 2010). Plants that live under strong UV can regulate pigmentation, different enzyme mechanisms, and photosynthesis in order to protect themselves from high radiation (*Franklin & Forster, 1997; Ekelund, 2000*).

### Potential impact of diffuse radiation on canopy productivity

**Figure 7:**
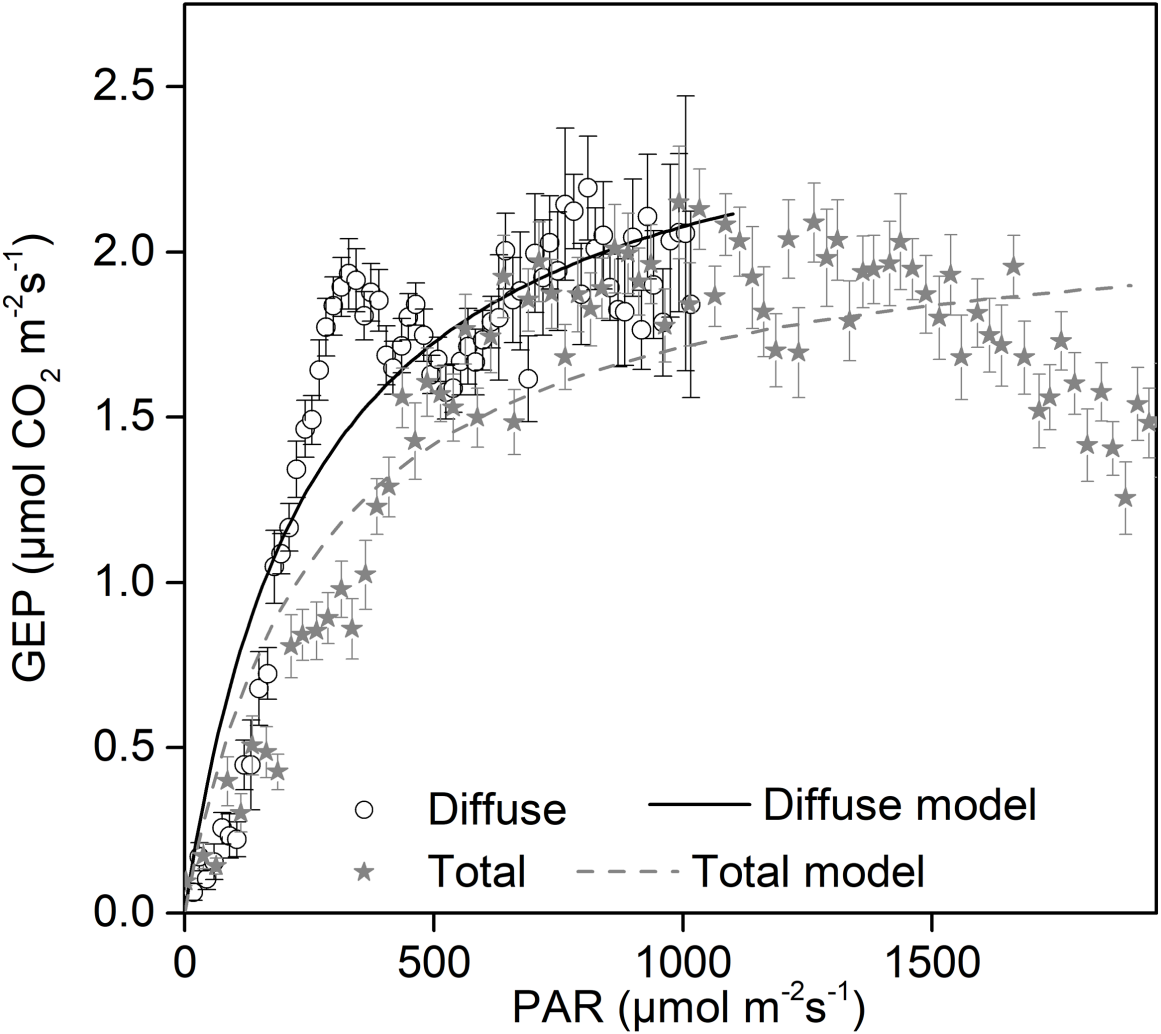
Daytime gross ecosystem production (GEP) as a function of diffuse photosynthetically active radiation (PAR_dif_) and of total photosynthetically active radiation (PAR_tot_) during mid-growing season (June–August) in 2014 and 2015. Half-hourly GEP was bin-averaged (a) by PAR increment of 50 µmol CO_2_ m^−2^ s^−1^. Bars indicate standard errors. Model parameters were fitted separately to diffuse and total radiation data, and the optimized model curves are shown as lines.

Increases in diffuse radiation play an important role in ecosystem GEP enhancement. The GEP peaked when the CI was around 0.5 (Fig. 4B) and when PAR_dif_ reached its maximum (Fig. 3D). The results revealed that vegetation reached its light saturation when PAR_dif_ reached its maximum. This CI range was consistent with those found in other studies on semi-arid steppes (*Zhang et al., 2011b*), grasslands (Bai et al., 2012), croplands (*Tong et al., 2014*), and forest ecosystems (*Gu et al., 1999*), but was higher than the values obtained in studies on short grass in semi-arid regions (*Jing et al., 2010*). We also observed that the light response curves differed under diffuse radiation and total radiation conditions (Fig. 7). The light-response curve *α* and P_max_ parameters are listed in Table 1. Two light response curves had similar trends, but the curves rose more steeply with light intensities under diffuse radiation. This result is consistent with previous studies (*Oliphant et al., 2011; Williams et al., 2014*). In the desert steppe ecosystem, a higher *α* value meant a higher potential light use efficiency, and a higher P_max_ meant a higher potential ecosystem production under diffuse radiation than under total radiation conditions. Those results indicated that the enhancement of canopy photosynthesis by diffuse radiation received by an ecosystem is more pronounced under cloudy conditions (*Gu et al., 1999; Alton et al., 2007a; Oliveira et al., 2007*). The effect of radiation changes, associated with an increase in clouds or scattered aerosols, on photosynthesis depends on the balance between total PAR reduction (which tends to reduce photosynthesis) and increased PAR diffusion (which tends to increase photosynthesis) (*Mercado et al., 2009*).

Due to a lack of measured PAR_dif_ data, our data was obtained using model calculations. We corrected the time zones according to Beijing time, but there is still some uncertainty about the simulated model. Other studies have shown that the model tends to overestimate PAR_dif_ when the fraction of diffuse PAR (fPAR_dif_) < 0.3 and fPAR_dif_ >0.9, and underestimates PAR_dif_ values in the fPAR_dif_ range of 0.3 and 0.9 (*Oliphant & Stoy, 2018*). Although the GEP model based on the diffuse beam partitioning model underestimated the observed GEP because of errors in the diffuse partitioning (*Lee et al., 2018)*, the diffuse beam partitioning model is still more accurate than other GEP models (*Zhou & Xin, 2019*).

We concluded that the increase of diffuse radiation under cloudy conditions has a positive impact on the GEP of the desert steppe. Further detailed analysis of how ecosystem diffuse radiation and other environmental factors affect carbon exchange processes are needed. Additionally, more data should be collected to distinguish the effects of aerosol, cloudiness, and UV on photosynthesis in mid-high altitude areas.

## Conclusion

Our results indicated that diffuse radiation could improve photosynthesis in the desert steppe ecosystem. The GEP peaked when the CI was around 0.5 with a maximum PAR_dif_. The P_max_ was 23.7% higher under cloudy skies than under clear skies. When the sky was cloudy, the PAR_dif_ became a positive influence on the desert steppe GEP. In addition, lower VPD (1 ≤ 1 kPa), lower T_a_ (T_a_ ≤ 20 °C), and non-stressed water conditions (REW ≥ 0.4) were more conducive to ecosystem photosynthetic enhancement under cloudy skies than under clear skies due to the comprehensive effects of VPD and T_a_ on stomatal conductance. Our study showed that diffuse radiation did encourage a potential increase in the production of desert steppe ecosystems in Northwest China. Further changes in sky conditions and diffuse radiation should be considered when modeling the GEP of desert steppe ecosystems.

##  Supplemental Information

10.7717/peerj.9043/supp-1 Supplemental Information 1 Daily rainfall in 2014, mm day^−1^

10.7717/peerj.9043/supp-2 Supplemental Information 2 Daily rainfall in 2015, mm day^−1^

10.7717/peerj.9043/supp-3 Supplemental Information 3 The first sample point soil volumetric water content in 2014 VWC soil volumetric water content in units of m3 m-3, port 1, 10 cm; port 2, 30 cm; port 3, 70 cm; port 4, 120 cm; port 5, 10 cm

10.7717/peerj.9043/supp-4 Supplemental Information 4 The second sample point soil volumetric water content in 2014 VWC soil volumetric water content in units of m3 m-3, port 1, 10 cm; port 2, 30 cm; port 3, 70 cm; port 4, 120 cm; port 5, 10 cm

10.7717/peerj.9043/supp-5 Supplemental Information 5 The third sample point soil volumetric water content in 2014 VWC soil volumetric water content in units of m3 m-3, port 1, 10 cm; port 2, 30 cm; port 3, 70 cm; port 4, 120 cm; port 5, 10 cm

10.7717/peerj.9043/supp-6 Supplemental Information 6 The first sample point soil volumetric water content in 2015 VWC soil volumetric water content in units of m3 m-3, port 1, 10 cm; port 2, 30 cm; port 3, 70 cm; port 4, 120 cm; port 5, 10 cm

10.7717/peerj.9043/supp-7 Supplemental Information 7 The second sample point soil volumetric water content in 2015 VWC soil volumetric water content in units of m3 m-3, port 1, 10 cm; port 2, 30 cm; port 3, 70 cm; port 4, 120 cm; port 5, 10 cm

10.7717/peerj.9043/supp-8 Supplemental Information 8 The third sample point soil volumetric water content in 2015 VWC soil volumetric water content in units of m^3^ m ^−3^, port 1, 10 cm; port 2, 30 cm; port 3, 70 cm; port 4, 120 cm; port 5, 10 cm

10.7717/peerj.9043/supp-9 Supplemental Information 9 Eddy covariance data and micrometeorological data of the desert steppe ecosystem in 2014

10.7717/peerj.9043/supp-10 Supplemental Information 10 Eddy covariance data and micrometeorological data of the desert steppe ecosystem in 2015

10.7717/peerj.9043/supp-11 Supplemental Information 11 Variables

## Additional Information and Declarations

### Data Availability

The following information was supplied regarding data availability: The raw measurements are available in the Supplementary Files.
