# Supplementary material for: Linking diffuse radiation and ecosystem productivity of a desert steppe ecosystem
Source: PeerJ. 2020 May 5;8:e9043. doi: 10.7717/peerj.9043 (PMC7207212; doi:10.7717/peerj.9043)
Supplement: Supplemental Information 11 [file peerj-08-9043-s011.docx]

The data variables and variable description:

The description of data variables and variables in the original data file is as follows:

Notes: The value -9999 is the error value in the system measurement when using the data.

**Rainfall_doy_（2014/2015）.xls**

| # | Parameter | Units | Description |
| --- | --- | --- | --- |
| 1 | Date | year-month-day | NA |
| 2 | DOY | NA | Day of year |
| 3 | Rainfall | mm | NA |

**yc_2_em50_（1/2/3）_（2014/2015）.xls**

| # | Parameter | Units | Description |
| --- | --- | --- | --- |
| 1 | Port1 | NA | 10 cm depth |
| 2 | SWC | cm^3^/cm^3^ | Soil water content |
| 3 | Temp | ℃ | Soil [temperature](javascript:;) |

**yc_2_flux_and_met（2014/2015）.xls**

| # | Parameter | Units | Description |
| --- | --- | --- | --- |
| 1 | date | year-month-day | NA |
| 2 | time | Hour:Minute | NA |
| 3 | daytime | True/False | 1 means true, and 0 means false |
| 4 | co2_flux | µmol s^-1^m^-2^ | Net ecosystem exchange |
| 5 | u* | m s^-1^ | Friction wind speed |
| 6 | S | W m^-2^ | Global solar radiation |
| 7 | PAR_tot | µmol s^-1^m^-2^ | Incident total photosynthetically active radiation |
| 8 | air_temperature | °C | Air temperature |
| 9 | RH | % | Relative humidity |
| 10 | VPD | KPa | Vapor pressure deficit |
